# Age-Dependent Accumulation of 8-Oxoguanine in the DNA and RNA in Various Rat Tissues

**DOI:** 10.1155/2013/303181

**Published:** 2013-04-29

**Authors:** Ben Nie, Wei Gan, Fei Shi, Guo-Xin Hu, Lian-Guo Chen, Hiroshi Hayakawa, Mutsuo Sekiguchi, Jian-Ping Cai

**Affiliations:** ^1^Graduate School, Wenzhou Medical College, University Town, Wenzhou, Zhejiang 325035, China; ^2^The Key Laboratory of Geriatrics, Beijing Hospital and Beijing Institute of Geriatrics, Ministry of Health, No. 1 Dahua Road, Dongdan, Beijing 100730, China; ^3^Graduate School, Chinese Academy of Medical Sciences and Peking Union Medical College, No. 9 Santiao, Dongdan, Dongcheng District, Beijing 100730, China; ^4^Department of Pharmacology, Wenzhou Medical College, University Town, Wenzhou, Zhejiang 325035, China; ^5^Frontier Research Center, Fukuoka Dental College, Fukuoka 814-0193, Japan

## Abstract

The relationship between the oxidative damage of nucleic acids and aging of animals was investigated by analyzing the nucleic acids derived from various tissue specimens of naturally aged Sprague-Dawley (SD) rats. For this purpose, we established an accurate and sensitive isotope-diluted LC-MS/MS method to determine the levels of 8-oxo-7,8-dihydro-2′-deoxyguanosine (8-oxo-dGsn) in DNA and 8-oxo-7,8-dihydroguanosine (8-oxo-Gsn) in RNA. An age-dependent increase in oxidative DNA and RNA damage was observed in the various organs examined, including the brain, liver, kidneys, and testes. Similar increases in the 8-oxo-dGsn and 8-oxo-Gsn contents were observed in three parts of the brain, the hippocampus, cerebral cortex, and cerebellum, among which, the values for the hippocampus were always the highest. When the oxidized guanosine metabolites were quantified with urine, a similar age-dependent increase was observed for both 8-oxo-dGsn and 8-oxo-Gsn. However, unlike the results of nucleic acid samples derived from the tissues, the amount of 8-oxo-Gsn was significantly higher compared to that of 8-oxo-dGsn, probably reflecting the fact that RNA degradation occurs more frequently than DNA degradation. Our finding indicates that the amount of urinary 8-oxo-Gsn could be considered as a biomarker for the sensitive measurement of oxidative stress and aging.

## 1. Introduction

Reactive oxygen species (ROS) are persistently generated in living cells, causing oxidative damage to cellular macromolecules, including proteins, lipids, and nucleic acids. Harman first proposed the free radical theory of aging in 1956 [1], and since then, there have been extensive ROS-related aging studies [2, 3]. More than 20 different kinds of base adducts have been found in DNA exposed to oxidative agents [4]. Since guanine has the lowest oxidation potential among the DNA bases, the guanine residues of nucleic acids are most readily oxidized by the hydroxyl radical (^•^OH) and singlet oxygen (^1^O_2_) [5]. It was supposed that there may be a link between the accumulation of oxidized guanine in nucleic acids and various age-associated pathological phenomena. Many studies have been focused on DNA [6–8] since DNA oxidative lesions must be repaired to maintain the genomic integrity. However, oxidative damage to RNA occurs more frequently than DNA, because RNA molecules are mostly single stranded, so that the bases are less protected by hydrogen bonding. Moreover, most of the mRNAs are not associated with chromatin and are distributed in the cytoplasm, closer to the site of ROS generation [9]. There is evidence that oxidized mRNA causes errors in translation, eventually leading to the production of abnormal proteins [10, 11]. Such abnormal proteins may be responsible for the occurrence of neurodegenerative diseases, such as Alzheimer's disease [12, 13].

Many methods have been developed to estimate the level of oxidative damage to nucleic acids. Immunohistochemistry has been used extensively to determine the amounts of 8-oxo-2′-deoxyguanosine (8-oxo-dGsn) in DNA and 8-oxoguanosine (8-oxo-Gsn) in RNA. The ELISA method has been applied for the quantification of oxidized guanosine in body fluid, including cerebrospinal fluid, plasma, and urine. Since the antibodies against 8-oxo-dGsn, used for immunochemistry and the ELISA assay sometimes exhibit cross-reactivity with other compounds. Chromatographic methods, such as HPLC-ECD, LC-MS/MS, and GC-MS, would provide more accurate means for analyzing the levels, with LC-MS/MS being the most reliable. Using this procedure, sample impurities can be eliminated during the HPLC phase, and all types of guanosine derivatives can be determined by tandem mass spectrometry based on their molecular weights. 

By using LC-MS/MS, we previously revealed an age-dependent accumulation of oxidative DNA and RNA damage in various organs of senescence-accelerated SAMP8 mice [14]. We also found an age-related increase in the levels of 8-oxo-dGsn and 8-oxo-Gsn in the leukocytes, plasma, and urine of *Macaca mulatta* [15]. However, in these animal models, the oxidative status of nucleic acids in different regions of the brain could not be determined due to the scant material available for the analyses. To overcome this difficulty, we used Sprague-Dawley rats in the present study. The mean life span of SD rats is 20.5 to 24.2 months [16], and the maximum life span varies from 967 (32.2 months) to 1000 days (33.3 months) for male animals [17, 18]. These rats would provide a better model to study the oxidative status of various tissues, especially those in subregions of brain. We applied the LC-MS/MS method to quantify oxidized guanosines in DNA and RNA in various tissues obtained from healthy SD rats at different ages. We therefore extended our analysis to plasma and urine samples to seek a useful marker of aging.

## 2. Materials and Methods

### 2.1. Chemicals

The 8-oxo-2′-deoxyguanosine (8-oxo-dGsn, >98% purity), 2′-deoxyguanosine (dGsn, >98% purity), guanosine (Gsn, 98% purity), and deferoxamine mesylate (DFOM) were purchased from Sigma-Aldrich (USA). The 8-oxo-guanosine (8-oxo-Gsn, >98% purity) was purchased from ALEXIS Biochemicals (San Diego, CA, USA). The [^15^N_5_]8-oxo-dGsn, [^15^N_5_]dGsn, and [^15^N_5_]Gsn were obtained from Cambridge Isotope Laboratories (Andover, MA, USA), and [^13^C, ^15^N_2_]8-oxo-Gsn was customized from Toronto Research Chemicals (Canada). Nuclease P1 and calf intestinal alkaline phosphatase were purchased from WAKO (Osaka, Japan) and NEB (USA), respectively. Ammonium acetate and methanol were of HPLC grade and were purchased from Fisher Scientific (USA). The water used for the determination process was deionized at 18.2 MΩ.

### 2.2. Instruments

A Shimadzu Prominence LC-20A was connected to an Applied Biosystem SCIEX QTRAP5500 triple quadrupole mass spectrometer with an ESI source controlled by the Analyst software program, version 1.5 (Sciex, Thornill, Canada). The column used was an Atlantis dC18 (2.1 × 150 mm, 5 *μ*m) obtained from Waters (USA). The mobile phase consisted of elution buffer A (10 mM ammonium acetate adjusted to pH 3.75 with acetic acid) and elution buffer B (methanol). The flow rate was 0.2 mL/min, and the column temperature was 30°C. The gradient of the mobile phase varied according to the type of sample (Supplementary Table 1 available online at http://dx.doi.org/10.1155/2013/303181). To reduce the contamination of the ion source, the early and late portions of the running sample were discarded.

Electrospray ionization was performed in the positive ion mode. The multiple reaction monitoring (MRM) mode was applied during quantification. Nitrogen was used for ion sources I and II, the curtain gas, and the collision gas. The nitrogen values were optimized to 55, 75, 30, and the medium value, respectively. The IonSpray Voltage was set to the Applied Biosystem SCIEX QTRAP5500. The temperature of the electrospray probe was 500°C (300°C for plasma samples). The optimized conditions for individual compounds are summarized in Supplementary Table 2.

### 2.3. Animals

Healthy male Sprague-Dawley rats were obtained from Vital River (Beijing, China). The animals were housed in a temperature-controlled room (24 ± 2°C) with a 12–12 h light-dark cycle, with six individuals of the same age per cage with free access to food and water. All the experiments were performed according to the National Institutes of Health Guide for the Care and Use of Laboratory Animals. Before sacrificing the animals, urine samples were collected in the evening for 2-3 consecutive days using the manual bladder palpation method as described previously [19] and stored at −40°C until they were analyzed. In the morning, the rats were sacrificed and blood was collected via postcaval exsanguination using Na_2_EDTA for anticoagulation. Plasma samples were immediately separated from whole blood by centrifugation (3000 rpm for 10 min at 4°C) and were frozen at −40°C until analysis. All organs, including the brain (the cerebellum, cerebral cortex, and hippocampus), heart, lungs, liver, kidneys, and testes, were immediately removed from the animals and quickly frozen in liquid nitrogen and stored at −80°C.

### 2.4. Preparation and Hydrolysis of Nucleic Acids

For the preparation of DNA, we followed the protocol recommended by the ESCODD [20, 21] with some modifications. After pulverization of an appropriate amount of tissue with liquid nitrogen, 1 mL of buffer A (10 mM Tris, 0.32 M sucrose, 5 mM MgCl_2_, 0.1 mM DFOM, and pH 7.5; 1% Triton X-100 added) was applied for cell lysis. Then, the reaction with RNase A and proteinase K was performed in 100 *μ*L of buffer B (10 mM Tris, 5 mM Na_2_EDTA, 0.1 mM DFOM, and pH 8.0). To prevent the artificial oxidation of samples, 200 *μ*L of NaI solution (40 mM Tris, 20 mM Na_2_EDTA, 7.6 M NaI, 0.1 mM DFOM, and pH 8.0) was applied, and the reaction was performed in the presence of 0.1 mM DFOM [22–24]. TRIzol (Invitrogen, USA) was used for extraction of total RNA according to the manufacturer's recommended protocol. In this case, 0.1 mM DFOM was added to the TRIzol reagent to prevent oxidation.

For the DNA analysis, 20 *μ*g of DNA was dissolved in 85 *μ*L of 0.1 mM DFOM solution and denatured by heating at 100°C for 3 min, followed by rapid chilling. To the DNA solution, 2 *μ*L of 20 ng/mL [^15^N_5_]8-oxo-dGsn and 2 *μ*L of 1 *μ*g/mL [^15^N_5_]dGsn were added as internal standards for the subsequent mass analysis. The DNA was hydrolyzed to nucleosides by incubation with nuclease P1 (dissolved in 0.3 M sodium acetate, 1 mM ZnSO_4_, and pH 5.3, at 1 U/*μ*L) at 37°C for 2 h and then with alkaline phosphatase (1 U/*μ*L) at 37°C for 1 h. The DNA hydrolysate in a total volume of 104 *μ*L was centrifuged at 12,000 ×g for 10 min at 4°C, and 80 *μ*L of the supernatant was used for the LC-MS/MS analysis. The hydrolysis of RNA was performed in the same manner, with some alterations in the reagent concentration. In short, 20 *μ*g of RNA was dissolved in 90 *μ*L of 0.1 mM DFOM solution, and 2 *μ*L of 50 ng/mL [^13^C, ^15^N_2_]8-oxo-Gsn and 2 *μ*L of 2.5 *μ*g/mL [^15^N_5_]Gsn were added as internal standards. Three microliters of nuclease P1 and 7 *μ*L of alkaline phosphatase were used for hydrolysis.

### 2.5. Preparation of Plasma Samples

The frozen plasma samples were thawed by incubating them at 37°C for 5 min. For 300 *μ*L of plasma, 6 *μ*L of 20 ng/mL [^15^N_5_]8-oxo-dGsn, and 6 *μ*L of 20 ng/mL [^13^C, ^15^N_2_]8-oxo-Gsn were added (keeping the ratio at 100 : 2 : 2). After a short vortex mixing, 900 *μ*L of acetonitrile was added, and the mixture was further mixed for 1 to 2 min and centrifuged (12,000 ×g for 10 min at 4°C) to isolate the supernatant. Acetonitrile was evaporated under a gentle steam of pure nitrogen. The residue was dissolved in 100 *μ*L of deionized water and centrifuged at 12,000 ×g for 10 min at 4°C, and 80 *μ*L of the supernatant was used for the LC-MS/MS analysis.

### 2.6. Preparation of Urine Samples

The frozen urine samples were thawed by incubating them at 37°C for 5 min. Each sample was divided into two aliquots, one for the LC-MS/MS analysis to quantify 8-oxo-dGsn and 8-oxo-Gsn and the other to quantify creatinine for normalization. For the oxidized guanosine analysis, 100 *μ*L of urine was mixed with 400 *μ*L of 30% methanol solution to obtain a 1 : 5 diluted urine sample. After centrifugation (12,000 ×g for 10 min at 4°C), 100 *μ*L of the supernatant was removed, and 2 *μ*L of 20 ng/mL [^15^N_5_]8-oxo-dGsn and 2 *μ*L of 20 ng/mL [^13^C, ^15^N_2_]8-oxo-Gsn were added. After gentle mixing, 80 *μ*L of the solution was used for the mass analysis.

For creatinine quantification, 100 *μ*L of urine was mixed with 900 *μ*L of 30% methanol to obtain a 1 : 10 diluted urine sample, which was then centrifuged (12,000 ×g for 10 min at 4°C). Ten microliters of the supernatant was injected into the HPLC-UV system (Waters, USA). For the quantitative analysis, a Waters Sunfire C18 (5 *μ*m, 4.6 × 250 mm) column was used with mobile phases A (20 mM ammonium acetate, pH 6.8, 95%) and B (acetonitrile, 5%). The UV detection wavelength was set at 233 nm, and the flow rate was set to 0.8 mL/min. The HPLC chromatograms are shown in Supplementary Figure 1.

## 3. Results

### 3.1. Oxidized Guanine Content in the DNA

**Figure 1:**
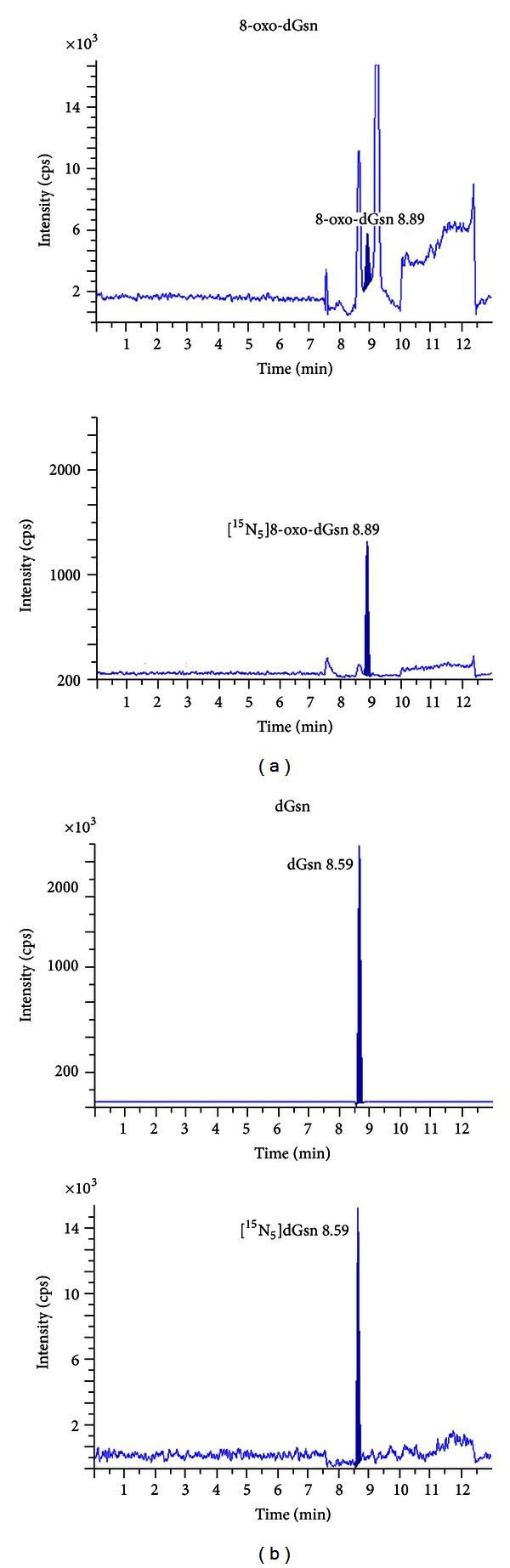
LC-MS/MS chromatograms of dGsn and 8-oxo-dGsn derived from DNA. DNA samples obtained from tissues were denatured and hydrolyzed to nucleosides by successive treatments with nuclease P1 and alkaline phosphatase. To each sample, [^15^N_5_]dGsn and 8-oxo-[^15^N_5_]dGsn were added to provide appropriate internal standards. The mixtures were applied to the LC-MS/MS system for quantification of the two types of deoxyguanosine, dGsn and 8-oxo-dGsn. The upper two figures show the elution patterns of DNA hydrolysates and the lower figures represent those for the internal standards.

To analyze the correlation between DNA oxidative damage and aging, we examined the 8-oxo-dGsn content of DNA samples derived from various tissues of the rats. To minimize the untoward effects of oxidation during the preparation of DNA, we adopted the ESCODD-recommended protocol [20, 21]. We further applied a metal chelator, deferoxamine methylate, to reduce the background level of oxidation [24]. The DNA was heat-denatured and hydrolyzed to nucleosides by successive treatments with nuclease P1 and alkaline phosphatase. To each sample, [^15^N_5_]dGsn and 8-oxo-[^15^N_5_]dGsn were added to provide appropriate internal standards. The mixtures were then applied to the LC-MS/MS system for quantification of the two types of deoxyguanosine, dGsn and 8-oxo-dGsn. As shown in Figure 1, dGsn and 8-oxo-dGsn were eluted at distinct positions and could be assayed without cross-contamination.

**Figure 2:**
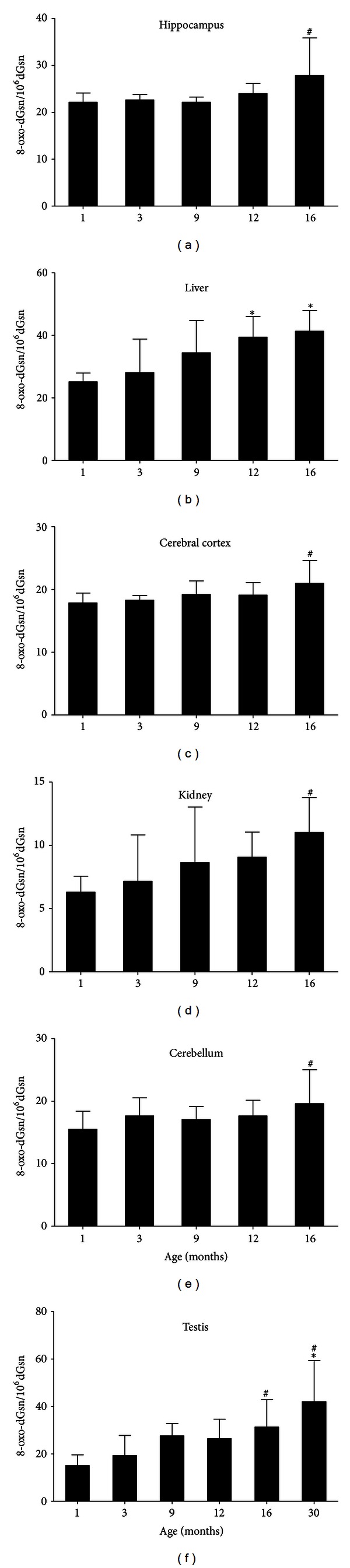
Age-related increases in the level of 8-oxo-dGsn/10^6^ dGsn in the hippocampus, cerebral cortex, cerebellum, liver, kidneys, and testes of SD rats. ^#^ *P* < 0.05, *P < 0.01, in comparison to one or several previous age groups. The data are presented as the means ± S.D. (*n* = 6 in each group).

The genomic DNA was prepared from the various tissue specimens obtained from the rats at different stages of growth and their contents of 8-oxo-dGsn were determined. The results are shown in Figure 2, in which the values are expressed as the numbers of 8-oxo-dGsn per 10^6^ residues of dGsn. To evaluate the effects of aging on the level of DNA oxidation, we determined the 8-oxo-dGsn contents in rats at five growth stages; 1, 3, 9, 12, and 16 months after birth (for the testes, the determination was extended to 30 months). In all of the organs examined, the 8-oxo-dGsn/10^6^ dGsn value increased gradually with the progression of age, and the maximum values were obtained for the oldest rats (16 or 30 months). A similar increase was observed in the three parts of the brain examined: the hippocampus, cerebral cortex and cerebellum, among which the hippocampus accumulated the highest levels of 8-oxo-dGsn. These results corroborate those of our previous immunohistochemical study using senescence-accelerated mice [25].

### 3.2. Oxidized Guanine in RNA

We extended our analyses to RNA samples obtained from the different age groups of rats. As was the case for DNA, precautions were taken to minimize the oxidation of the materials during the preparation of RNA as well as the enzymatic digestion to nucleosides. Under the conditions used for liquid chromatography, Gsn and 8-oxo-Gsn were eluted at distinct positions, which were also different from those of their deoxyribonucleoside counterparts. To ensure accurate determinations, we included ^15^N-substituted ribonucleosides as internal standards.

**Figure 3:**
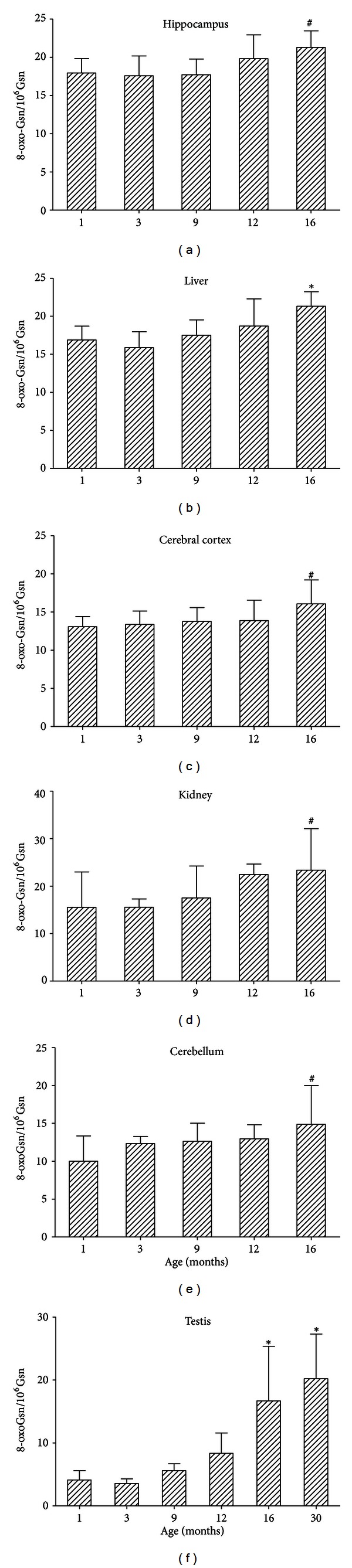
Age-related increases in the level of 8-oxo-Gsn/10^6^ Gsn in the hippocampus, cerebral cortex, cerebellum, liver, kidneys, and testes of SD rats. ^#^ *P* < 0.05, *P < 0.01, in comparison to one or several previous age groups. The data are presented as the means ± S.D. (*n* = 6 in each group).

Figure 3 shows the 8-oxo-Gsn contents of RNAs derived from various tissues of SD rats at different ages. The values were expressed as the 8-oxo-Gsn/10^6^ Gsn to estimate the levels of the oxidative damage to RNA. The patterns of age-related changes in the 8-oxo-Gsn content were largely the same for all the organs examined. An age-dependent increase in the oxidative RNA damage was also observed in the three parts of the brain, with the hippocampus again exhibiting the highest values at all of the ages examined. These results are in accord with our previous finding with SAMP8 mice, in which substantial amounts of 8-oxoguanine were present in the hippocampi of aged animals [26].

### 3.3. The Level of Oxidized Guanosine in Urine

**Figure 4:**
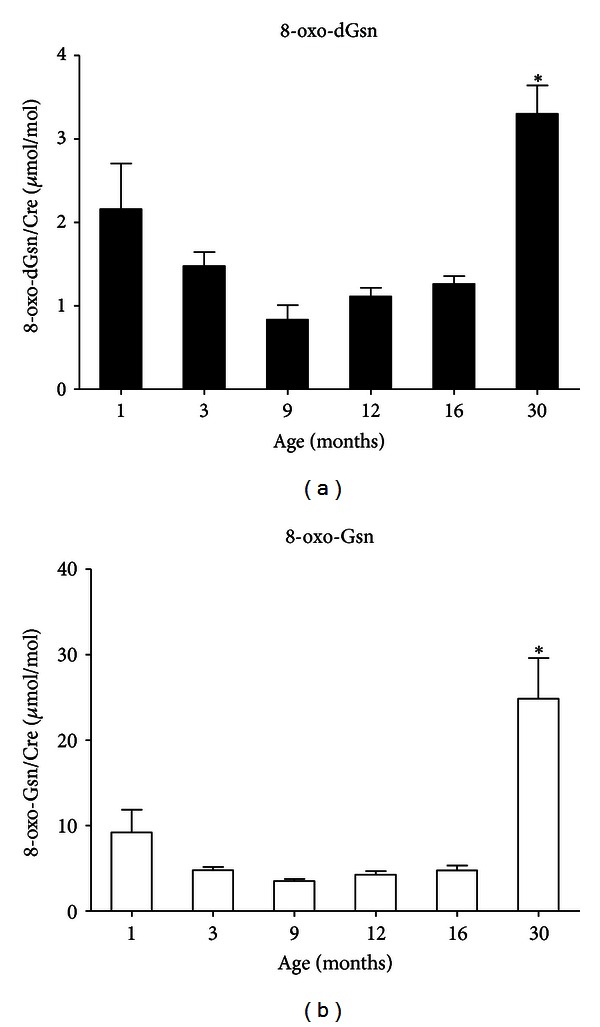
The LC-MS/MS measurement of the concentrations of 8-oxo-dGsn and 8-oxo-Gsn in the urine of SD rats. (a) Age-related increases in the level of 8-oxo-dGsn in the urine. (b) Age-related increases in the level of 8-oxo-Gsn in the urine. *P < 0.01, in comparison to one or several previous age groups. The data are presented as the means ± S.D. (*n* = 6 in each group).

We also applied the LC-MS/MS method to estimate the amount of the oxidized forms of guanine derivatives, 8-oxo-dGsn and 8-oxo-Gsn, in urine. Since the volumes of urinary samples varied and their content was affected by the renal glomerular function, we normalized the oxidized guanosine level to the creatinine level. These values were determined using samples obtained from animals at different ages, and the results are summarized in Figure 4. Although the amounts of 8-oxo-dGsn and 8-oxo-Gsn were still relatively low at age of 16 months, the values increased significantly in animals by the age of 30 months. It was noted that 30 months after birth, the content of 8-oxo-Gsn was approximately four times that of 8-oxo-dGsn (Table 1).

When an analysis was performed using plasma, a similar but less striking difference in the contents of the two types of oxidized nucleosides was obtained (Table 1). Since the concentrations of 8-oxo-dGsn and 8-oxo-Gsn in plasma are extremely low, it was difficult to make accurate measurements using plasma samples. In this respect, the results with the urinary samples may be considered to provide a better biomarker for oxidative stress, at least in rats.

## 4. Discussion

All living cells constantly generate oxygen free radicals, and it is unavoidable that the components of nucleic acids are thus oxidized. An oxidized form of guanine, 8-oxoguanine, formed in DNA would yield a profound effect, since it can pair with adenine during DNA replication, leading to GC→TA transversion mutations [27]. Oxidized mRNA may impair the translation fidelity, causing the formation of abnormal proteins [10, 11]. To prevent such outcomes, organisms possess elaborate defense mechanisms, including those for eliminating oxidized precursor nucleotides and for repairing oxidized DNA. The MutT protein of *Escherichia coli* is able to hydrolyze 8-oxo-dGTP and 8-oxo-dGDP to nucleoside monophosphate, thereby preventing the misincorporation of oxidized guanine into DNA [28]. The same mechanism for eliminating unfavorable substrates from the precursor pool may also function for RNA synthesis. The *E. coli* MutT protein cleaves oxidized forms of RNA precursors, 8-oxoGTP and 8-oxoGDP, as efficiently as the deoxyribonucleotide counterparts [28]. In mammalian cells, multiple species of proteins appear to be involved in preventing the misincorporation of 8-oxoguanine into DNA and RNA. The human proteins, MTH1, MTH2 and NUDT5, are structurally similar to the *E. coli* MutT [29–31], and both MTH1 and NUDT5 have the ability to degrade 8-oxoGTP and 8-oxoGDP, ensuring to block the misincorporation of 8-oxoguanine into RNA [30]. Regarding the repair of oxidative lesions in DNA, two glycosylases, MutM and MutY, function in *E. coli*. The MutM protein eliminates 8-oxoguanine from double-stranded DNA, whereas the MutY protein removes adenine from the mutagenic 8-oxoG:A pair [32]. The eukaryotic homologues of MutM and MutY have been identified as OGG1 and MYH, respectively, and these proteins appear to serve the same purpose in mammalian cells [33, 34]. However, this type of excision repair mechanism is not functional for RNA, due to its single-stranded structure; OGG1 (MutM) only recognizes 8-oxoguanine when paired with cytosine [9]. It has been hypothesized that oxidized RNA may be recognized by certain protein factors and then subjected to degradation. Oxidized mRNA appears to be especially susceptible to proteasome-mediated degradation [11].

In our current study using the LC-MS/MS, we found that the accumulation of oxidative lesions increased with age in both DNA and RNA samples derived from various organs in SD rats. These cells obviously suffered from higher oxidative stress as a result of aging. Compared to other organs, the liver appears to bear a higher level of oxidative stress, as observed by the 8-oxo-Gua content of both the DNA and RNA. This observation may be attributed to the fact that the liver is a vital metabolic organ and would produce a large amount of ROS. The significantly increased oxidative damage to the nucleic acids observed in the testes might be due to degenerative changes of the genital system in old age. Among the three brain regions examined, the hippocampus exhibited the highest level of accumulation of oxidative lesions in both DNA and RNA at all ages. It can be inferred that oxidative lesions may be a cause of impaired cognition in some age-related neurodegenerative diseases such as Alzheimer's disease.

We supposed that RNA may have higher levels of oxidative lesions compared with DNA, due to its single-stranded structure, lack of protection by histones, and its cellular distribution close to the site of ROS generation. However, in all of the organs examined, except for the kidneys, there were higher levels of oxidized guanine in the DNA rather than in the RNA. This might be related to the fact that the half-life of oxidized RNA is much shorter than that of oxidized DNA. If this is the case, a loss of specific enzymes to remove oxidized RNA would lead to the accumulation of abnormal proteins, potentially leading to diseases such as AD. 

We extended our analyses to the blood plasma and urine samples to evaluate the levels of oxidative stress in rats at different growth stages. Our results indicated that the levels of 8-oxo-dGsn and 8-oxo-Gsn in plasma range from 0.01 to 0.1 nM, which is too low to be evaluated using the current analytical instruments and methods. Urine, on the other hand, contains a higher level of oxidized guanosine, thus providing a useful biomarker for aging. Age-related increases in the amounts of 8-oxo-dGsn and 8-oxo-Gsn were evident when the urine samples from rats at various growth stages were analyzed. Interestingly, the levels of 8-oxo-Gsn in the urine were higher than those of 8-oxo-dGsn, probably reflecting the fact that oxidized RNA is generally subjected to degradation, whereas in DNA, only the damaged regions is replaced. It is conceivable that RNA molecules may thus be more susceptible to oxidative damage than DNA, since most regions of RNA are single stranded. As a result, a relatively large amount of 8-oxo-Gsn would be released into the bodily fluids, including urine. The 8-oxoguanine in urine can be considered to be a sensitive biomarker for evaluating the level of oxidative stress affecting the whole body. Although the level of 8-oxo-dGsn in the urine has been widely investigated as a biomarker for aging [35], our data indicate that the urinary 8-oxo-Gsn could be a more sensitive and reliable biomarker for aging and/or oxidative damage.

## Supplementary Material

The Supplementary Material provide the detailed information about the experimental procedure, including "elution conditions for each sample", "Mass conditons for each compound", and "HPLC chromatograms of creatinine".

## Figures and Tables

**Table 1:** The levels of the two types of oxidized guanosine in the urine and plasma of 30-month-old SD rats.

Sources	Forms of guanosine	Concentration of oxidized guanosine (nM)	Ratio of 8-oxo-Gsn to 8-oxo-dGsn
Urine	8-oxo-dGsn	15.42	3.75
	8-oxo-Gsn	57.87	

Plasma	8-oxo-dGsn	0.058	1.92
	8-oxo-Gsn	0.111	
