# Supplementary material for: Age-Dependent Accumulation of 8-Oxoguanine in the DNA and RNA in Various Rat Tissues
Source: Oxid Med Cell Longev. 2013 Apr 29;2013:303181. doi: 10.1155/2013/303181 (PMC3657452; doi:10.1155/2013/303181)

Supplementary Table 1. Gradient of the mobile phase for each kind of samples

Samples	Different ratio of elution A and B in gradient (%)		
	A: 95, B: 5	A: 50, B: 50	A: 95, B: 5
DNA	0-3 min	3.01-4 min	4.01-13 min
RNA	0-3 min	3.01-4 min	4.01-13 min
Urine	0-2 min	2.01-3 min	3.01-13 min
Plasma	0-2 min	2.01-3 min	3.01-13 min

Supplementary Table 2. Conditions for individual compound

Compound	MRM pairs	Internal Standard MRM pairs	DP	CE	CXP	EP
8-oxodGsn	284.1/168.1	289.1/173.1	50	17	13	10
dGsn	268.1/152.1	273.1/157.1	180	47	14	10
8-oxoGsn	300.1/168.1	303.1/171.1	50	25	7	10
Gsn	284.1/152.1	289.1/157.1	250	35	12	10

Supplementary Fig. 1. HPLC chromatograms of creatinine standard (A) and urine sample (B)

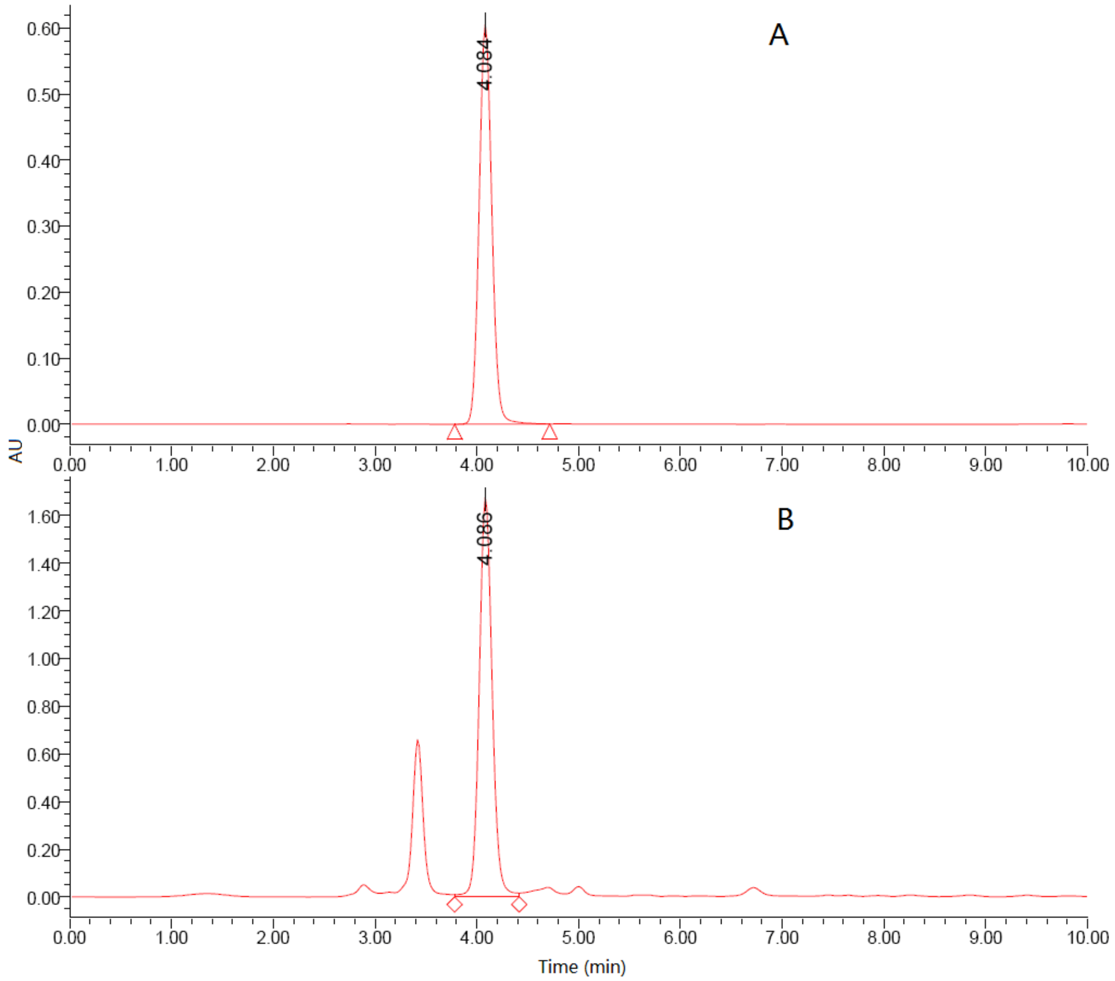

Supplement: Supplementary file 1 — The Supplementary Material provide the detailed information about the experimental procedure, including "elution conditions for each sample", "Mass conditons for each compound", and "HPLC chromatograms of creatinine". [file 303181.f1.pdf]
